# A variance-guided resampling framework for better K-medoids clustering

**DOI:** 10.1016/j.mex.2026.104198

**Published:** 2026-10-08

**Authors:** Kariyam, Feri Wijayanto, Edy Widodo

**Affiliations:** a Department of Statistics, Universitas Islam Indonesia, Jl. Kaliurang km 14.5, Sleman, Yogyakarta, 55584, Indonesia; b Department of Informatics, Universitas Islam Indonesia, Jl. Kaliurang km 14.5, Sleman, Yogyakarta, 55584, Indonesia

**Keywords:** ResConeK, Block-based k-medoids, Resampling, Representative object blocks, Cluster homogeneity, Cluster validity

## Abstract

One of the limitations of the k-medoids clustering algorithm is its susceptibility to local optimization and the potential for identical or similar objects to be assigned to different initial clusters. This study proposes ResConeK method, a modification of the block-based k-medoids algorithm that integrates a resampling strategy into the selection of representative object blocks formed according to similar variances. The method is validated using six external clustering criteria—Rand Index, Adjusted Rand Index, Hubert Similarity Statistic, Jaccard Similarity, Fowlkes–Mallows Index, and Rogers–Tanimoto Index—together with an internal criterion based on cluster homogeneity. Performance is benchmarked against four clustering methods: fast *k*-medoids, block-based *k*-medoids, Ward's method, and hierarchical clustering with complete linkage. Experiments show that the average validity score of ResConeK is comparable to all benchmark methods on three and four clusters, and significantly higher than block-based k-medoid on seven clusters and has comparable homogeneity for real data.

The proposed algorithm:

• Applicable to all data types, with greater benefits for datasets containing many identical objects or objects exhibiting similar distance variance.

• Introduces a resampling strategy on representative object blocks within the *k*-medoids framework.

• Reduces the risk of local optima and improves cluster homogeneity and stability.

## Graphical abstract

**Figure:**
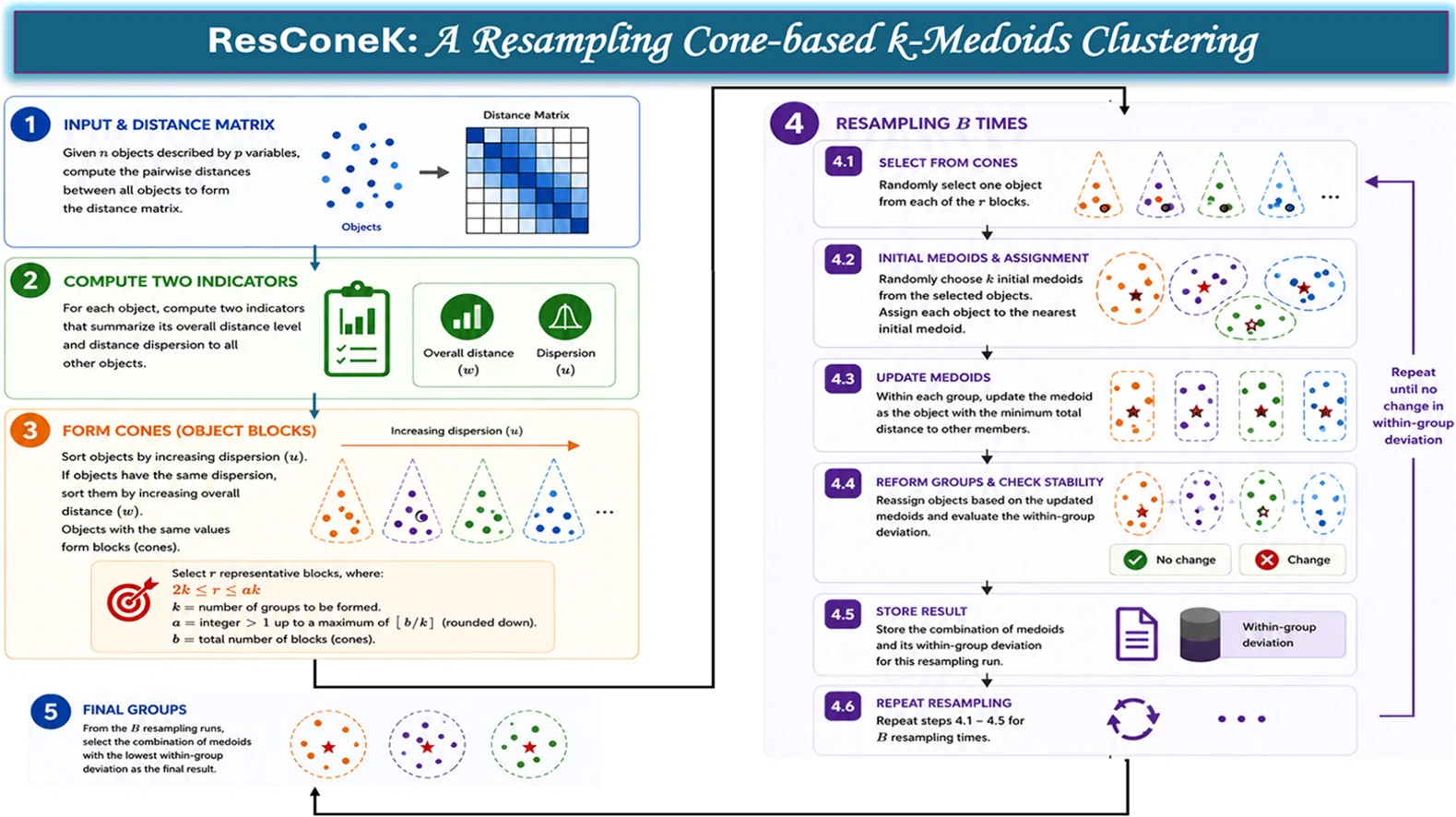

## Specifications table

**Table:** 

Subject area	Mathematics and Statistics
More specific subject area	Medoid-based Clustering
Name of your method	ResConeK: A Resampling Cone-Based k-Medoids Clustering
Name and reference of original method	H.S. Park, C.H. Jun, A simple and fast algorithm for k-medoids clustering. Expert System with Applications, 36 (2) (2009) 3336–3341. 10.1016/j.eswa.2008.01.039 Kariyam, Abdurakhman, Subanar, H. Utami, A.R. Effendie, Block-based k-medoids partitioning method with standardized data to improve clustering accuracy, Mathematical Modelling of Engineering Problems, 9 (6) (2022) 1613-1621. 10.18280/mmep.090622
Resource availability	▪ The benchmark datasets used in this study are publicly accessible through the UCI Machine Learning Repository, https://archive.ics.uci.edu/

## Background

Clustering algorithms can be agglomerative (hierarchical) with an unknown number of groups and partition-based (non-hierarchical) with a specified number of groups. The non-hierarchical k-medoids technique was developed to overcome outliers that are not optimal when solved with k-means [[1], [2], [3]]. One of the issues in k-medoids is related to the initial medoid selection technique (initialization), and various studies have been conducted on this issue [[4], [5], [6], [7], [8], [9]].

In 2009, Hae-Sang Park and Chi-Hyuck Jun proposed the Simple and Fast *K*-Medoids (SFKM) algorithm [6]. One limitation of SFKM arises when one or more identical objects are selected as initial medoids. Because identical objects may be assigned as different initial medoids, the algorithm can produce one or more empty initial clusters [7]. To address this issue, Budiaji and Leisch proposed the Simple K-Medoids (SKM) algorithm in 2019 by modifying the initialization procedure. In SKM, if an object selected as an initial medoid has identical counterparts, those identical objects are temporarily retained in their respective initial clusters during the initialization stage [7]. Although this modification prevents empty initial clusters, identical objects may still belong to different initial clusters before the iterative optimization process, even though they are likely to be assigned to the same cluster in the final solution [8].

Kariyam et al. developed the Block Distance-based K-Medoids (BKMD) partitioning method [8,9], which focuses on overcoming the limitations of the SFKM and SKM methods. The BKMD method uses indicators of the same number and variance of data on several of observation variables to form a block of objects. The initial medoid in the BKMD method is the first object in the first k blocks. This strategy prevents identical objects from being simultaneously selected as initial medoids and avoids the formation of empty initial clusters. However, despite these improvements, the BKMD algorithm remains susceptible to convergence to local optima.

To address the above limitation, this paper proposes ResConeK, a modification of the block-based k-medoids (BKMD) algorithm designed to reduce the likelihood of convergence to local optima. Building on BKMD's construction of representative object blocks, formed by grouping objects with similar distance variance, ResConeK replaces BKMD's deterministic initial-medoid selection with a resampling strategy applied within these blocks. Multiple candidate medoid combinations are generated across resampling iterations, and the combination yielding the minimum within-cluster distance is retained as the final solution. Through this modification, ResConeK aims to produce more homogeneous clusters than the original BKMD algorithm. The effectiveness of the proposed method is evaluated using six external clustering validity indices and one internal homogeneity criterion and is compared with four clustering methods on three artificial datasets and six real-world datasets from the University of California, Irvine (UCI) repository.

## Method details

The three main steps in cluster analysis are (1) selecting variables, standardization, and selecting distance functions; (2) selecting algorithms and their parameters; and (3) selecting validation techniques [10]. Data standardization is aimed at equalizing the size of data variability or adjusting the magnitude of scores and relative weights of variables in the formation of proximity measures [11,12]. Research related to clustering algorithms has been widely performed whether related to large-dimensional data [13,14], mixed data types [15,16], various new clustering algorithms [17], and various clustering applications both in the field of civil engineering [18], the environmental field [19], the economic field [20], and many more. Likewise, many themes related to the criteria for estimating group size as the third main stage have also been developed. Some criteria are formed using the concept of homogeneity in groups [21,22], based on homogeneity and heterogeneity [23,24], and many others. Grouping techniques or algorithms, as the next step in group analysis, the focus of this research, remain an interesting topic to be explored.

### ResConeK: a resampling cone-based k-medoids clustering algorithm (proposed method)

A novel non-hierarchical partitioning method, termed ResConeK, is introduced herein. This algorithm was developed with the concept of determining the initial medoid based on resampling on a data set (r objects) representing the object blocks. The ResConeK partitioning method is a modification of the BKMD method and still uses two BKMD indicators in forming object blocks. This is because the BKMD method guarantees that identical or nearly identical objects will be in the same initial group [8,9]. In BKMD, the initial medoid is only determined once by taking the first data in the first k blocks. We modified BKMD by changing the method of determining the initial medoid which is done by resampling B times. Next, the combination of resampled data that produces the smallest total deviation value in the group is determined as the final medoid.

The detailed steps of the new ResConeK algorithm that we propose are as follows.

(1) Suppose $x_{i}$ (i=1,2,⋯,n),is object observed on p variables. Determine the distance matrix, $D(x_{i},x_{j}),$ for all possible pairs of objects $x_{i}$ and $x_{j}$ with i=j=1,2,⋯,n.
(2) For each object $x_{i}$ (i=1,2,⋯,n) two indicators $u_{i}$ and $w_{i}$ are computed as follows:
  (1) $$w_{i}=\;\sum_{j=1}^{n}D\left(x_{i},x_{j}\right)$$
  (2) $$u_{i}=\frac{\sum_{j=1}^{n}{\left(D\left(x_{i},x_{j}\right)-{\bar{d}}_{i}\right)}^{2}}{n-1}$$

with

(3) $${\bar{d}}_{i}=\frac{w_{i}}{n}.$$

3) Sort variance $u_{i}$ (i=1,2,⋯,n)in ascending order from smallest to largest. If any objects have the same $u_{i}$ value, sort the object blocks with that value in ascending order based on the $w_{i}$ value. Thus, there will be up to b(b≤n) object blocks with the same $u_{i}$ and $w_{i}$ values, as if this block forms a cone. Determine set of r=ak(2k≤r≤b) blocks with the highest frequency. The value of k is the number of groups to be formed. The value of a is an integer greater than two up to a maximum of b/k (rounded down). In this study, a coefficient value of 4 was use for datasets with two groups, and 3 for datasets with more than two groups.
4) Resampling B times

  (i) Randomly select one object from r blocks. Determine k initial medoids by randomly selecting from the set r representative object blocks. Then determine the k initial group members based on the distance of each object to its nearest initial medoid.
  (ii) Update the medoid in each group $\left(m_{g}=\mathrm{medoid}\left(G\right)\right)$ based on the objects with the minimum sum of distances (equivalent to the average distance) to other members in the initial group, as shown in Eq. (4),
    (4) $$\mathrm{medoid}\left(G\right)≔\underset{x_{i}\in G}{\arg\;\min}\sum_{x_{j}\in G}D\left(x_{j},x_{i}\right).$$
  (iii) Use the results of Eq. (4) to create a new group. Repeat the medoid updates (candidate final medoid) until the Within-Group Deviation Sum, SDW(k) in Eq. (5) does not change, thus forming the final candidate group.
    (5) $$\mathrm{SDW}\left(k\right)=\sum_{g=1}^{k}\sum_{i=1}^{n_{g}}D\left(x_{\mathrm{gi}},m_{g}\right)$$
    5) Set the combination of medoids updates from B resampling times that produces the lowest SDW(k)as the final group.

The fundamental difference between the ResConeK algorithm and the BKMD clustering method, introduced by Kariyam et al. [8] as part of their dissertation [9], lies in the incorporation of a B-times resampling strategy applied to variance-based object partitioning. In the BKMD method, the initial medoids are deterministically selected as the first objects from each of the first (k) partitions. For example, in the BKMD method, when forming four clusters, the initial medoids are chosen as the first objects in partitions (P1), (P2), P(3) and (P4) such in Fig. 1. Consequently, other objects within the same partition, as well as objects from different partitions, are never considered as candidate initial medoids. This deterministic initialization increases the likelihood of converging to a local optimum.

**Fig. 1:**
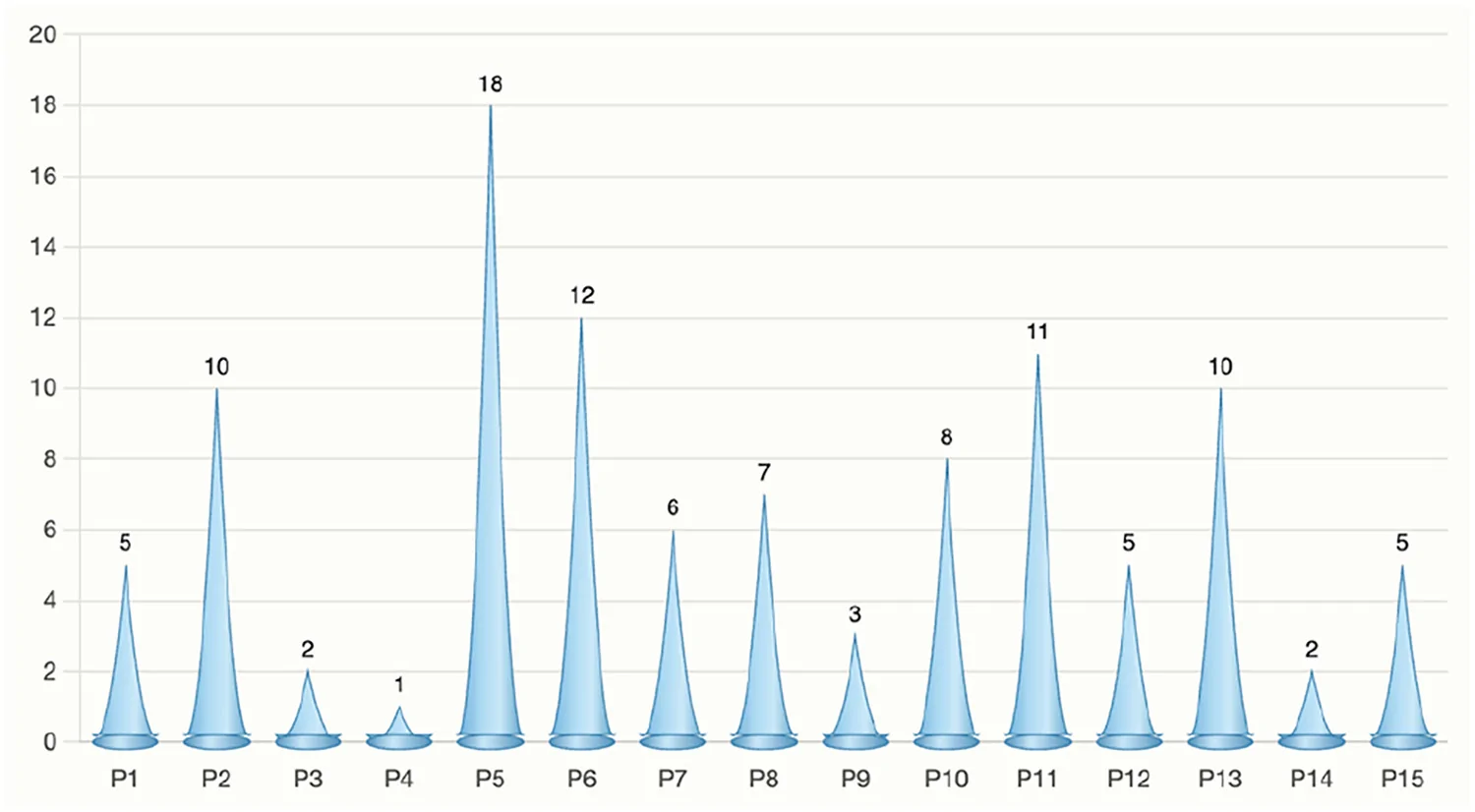
Illustration of b sets of objects with similar variance and number of data.

To address this limitation, the proposed ResConeK algorithm introduces two key modifications. First, instead of selecting fixed representative objects, the initial medoids are randomly sampled from the (r) partitions with the highest frequencies. For example, in the ResConeK algorithm, if we are going to form three groups with the value of a is three, then based on Fig. 1, the nine blocks are P5, P6, P11, P2, P13, P10, P8, P7, and P1 each containing 18, 12, 11, 10, 10, 8, 7, 6 and 5. Second, the initialization procedure is repeated (B) times using resampling over these selected partitions. Among the resulting candidate solutions, the medoid combination that produces the minimum within-group deviation sum, (SDW(k)), is selected as the final clustering solution. The recommended parameter B is between 10 to 20 times. By exploring various initialization configurations while maintaining a variance-based partition structure, ResConeK substantially mitigates the possibility of local optimization.

The complexity of the ResConeK algorithm remains of order n², the same as BKMD, because both calculate the full distance matrix and update the medoid based on the sum of the distances to all group members. The total complexity of the ResConeK method is $O(n^{2}\left(p+\mathrm{BT}\right))$ because there are B times of resampling and each resampling is carried out in T iterations to achieve convergence. ResConeK utilizes the cone block formation stage as a filtering step before resampling. Because objects within a cone block empirically behave as representations of each other, a large number of resamplings are not required to obtain a stable medoid estimate, this is why B = 10–20 times. With a fixed and relatively small B (10–20) and a practically small T (medoid update iterations until convergence per resampling), the BT factor in ResConeK's complexity, $O(n^{2}\left(p+\mathrm{BT}\right)$, acts as a controlled constant, not a variable that grows with n. Thus, in terms of large-order (big-O with respect to n), ResConeK and BKMD are in the same complexity class, O(n²).

## Method validation

The performance evaluation was conducted using an empirical approach. In this approach, six external cluster validation measures were compared across five clustering techniques. Three artificial data scenarios were employed to test these techniques, after which they were applied to six real-world datasets. The clustering techniques considered were Simple and Fast k-medoids (SFKM) [6], distance block-based k-medoids (BKMD) [8,9], hierarchical agglomerative clustering with complete linkage, and Ward’s method [2]. The external validation measures utilized were *Rand Index (RI), Adjusted Rand Index (ARI), Hubert Similarity Statistics (HSS), Jaccard Similarity (JS), Fowlkes-Mallows Index (FMI)* and *Rogers*-*Tanimoto Index (RTI)* [2,10].

Three artificial datasets were designed: three two-variable groups, four three-variable groups, and seven two-variable groups. Each scenario was repeated fifty times, and the average external criterion index was compared. The six datasets from the UCI repository consist of breast cancer [25], primary tumor [26], heart disease [27], iris [28], wine [29] and soybean small [30]. The Breast Cancer (BC) dataset consists of 683 breast tissue samples with 9 numerical variables related to tissue characteristics. The data are grouped into two categories: patients diagnosed with benign or malignant breast cancer types [25]. Primary tumor data relates to determining the location of the tumor. This study used only four of the 22 groups and 65 non-missing data points. All 17 features in this dataset represent characteristics of tumor patients [26]. The Heart Disease (HD) dataset comprises 303 instances with 13 mixed-type variables, including 3 binary, 5 categorical, and 5 numerical variables, grouped into two categories. This dataset is used for heart disease classification analysis whether a subject has or does not have heart disease. The features are related to risk factors and symptoms of heart disease, such as blood pressure, cholesterol levels, and others [27].

The Iris dataset consists of 50 samples from each of three Iris species (Iris setosa, Iris virginica, and Iris versicolor). Four features are measured for each sample: the length and width of sepals and petals, in centimeters [28]. The Wine dataset contains the results of chemical analysis of wines grown in the same region in Italy but derived from three different cultivars. The analysis measured the quantities of 13 constituents found in each of the three wine types. This dataset includes 178 instances with 13 numerical variables, grouped into three categories [29]. The Soybean Small dataset consists of 47 soybean plants described by 35 non-metric disease-related variables, grouped into four categories. The objective of this dataset is to classify plant diseases based on symptoms and other features [30].

### Artificial data

The first artificial data is formed in three groups, and each object consists of two numerical variables. Each group of size 50 is generated from a normal distribution with the centers of each group set to (10, 10), (15, 15), and (15, 7). The standard deviation for each group is one. An example, the distribution of artificial data is shown in Fig. 2. The average values of the six external validity indices for each clustering method are shown in Fig. 3.

**Fig. 2:**
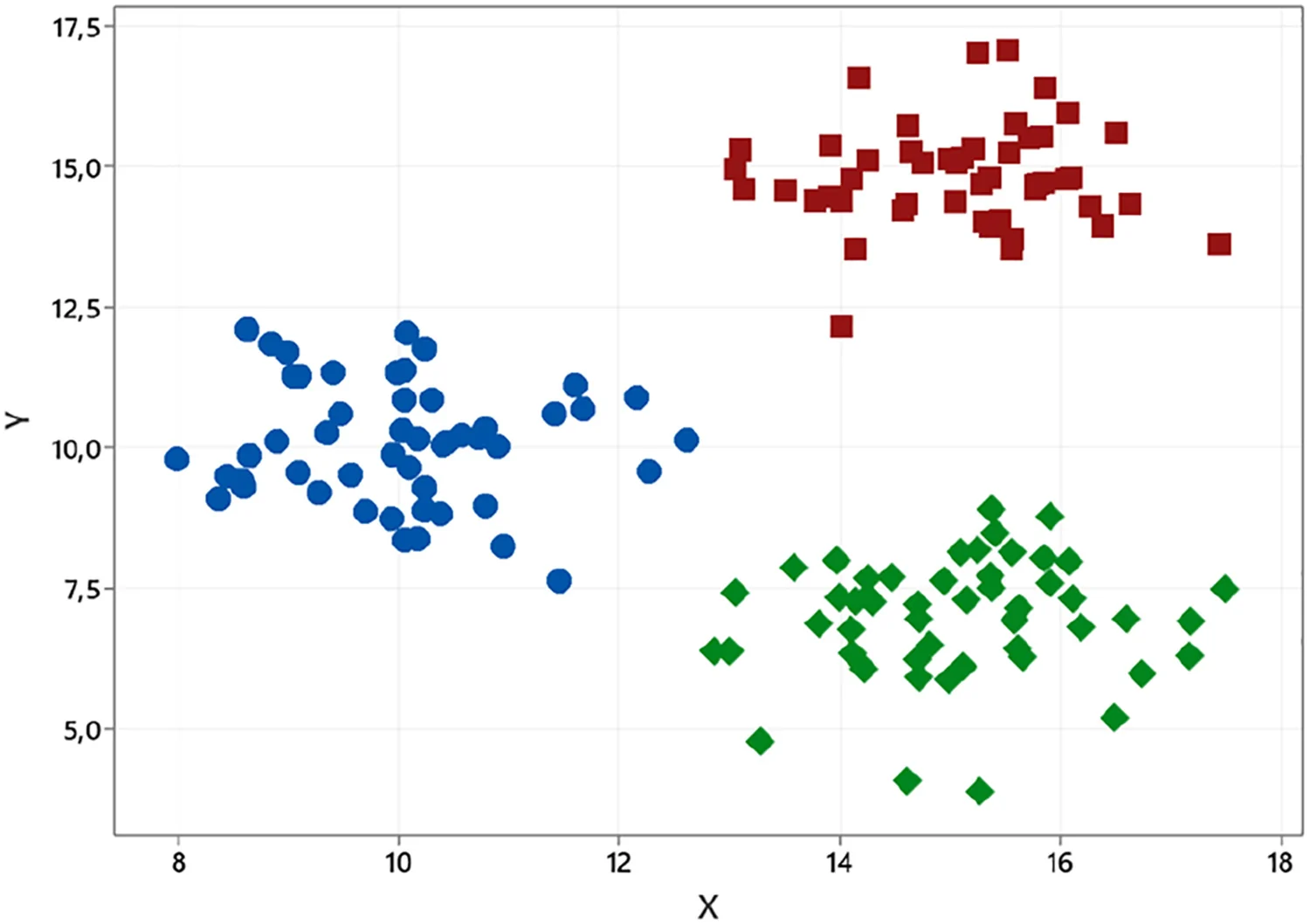
Artificial data consisting of three groups.

**Fig. 3:**
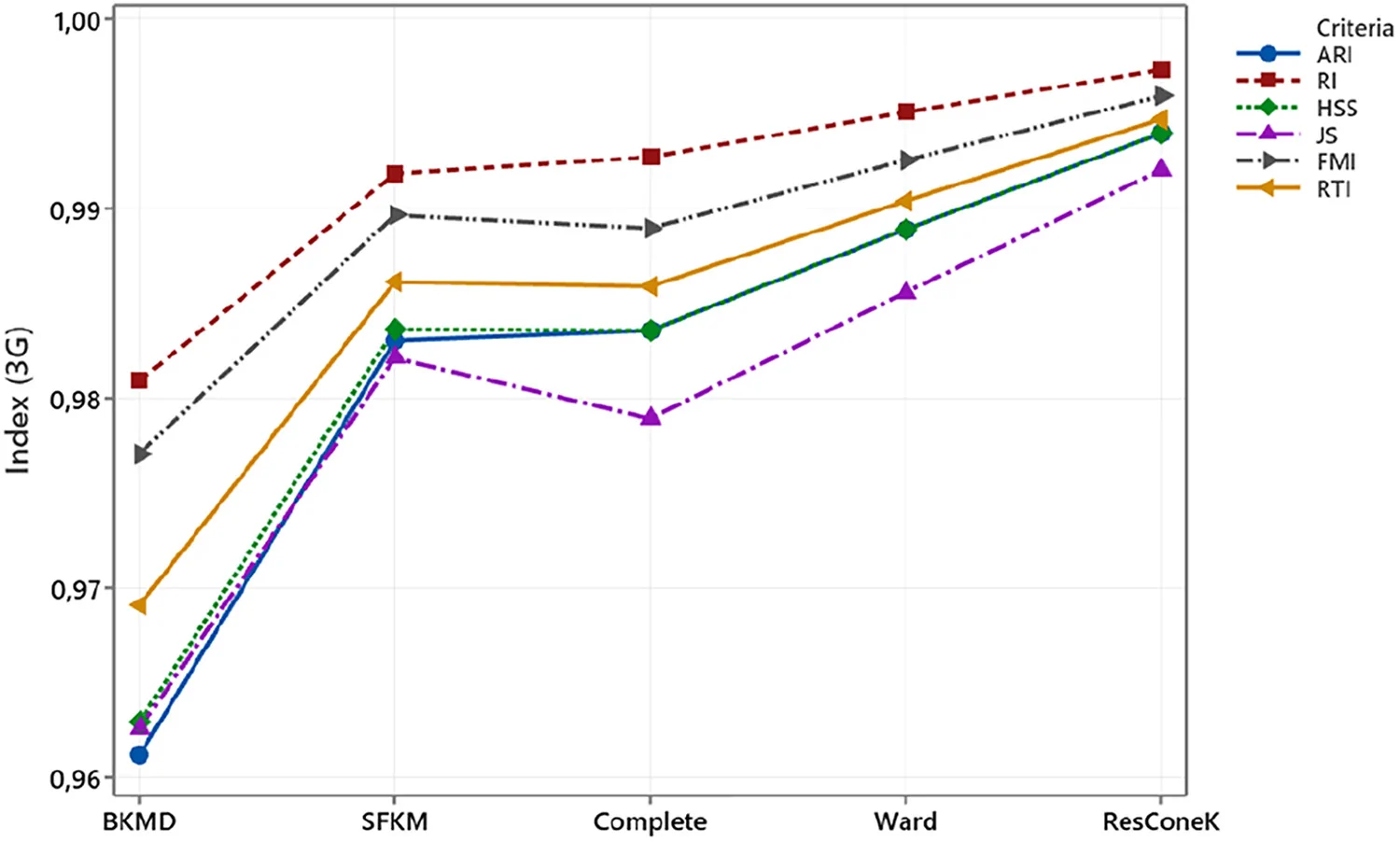
Comparison of external validity indices of three-group artificial data.

It is shown in Fig. 3 that, in the case of the first artificial dataset illustrated in Fig. 2, descriptively, a consistent ordering pattern is observed across all six indices. BKMD and SFKM record the lowest mean scores, followed by a moderate increase for Complete-linkage and Ward's method, with ResConeK attaining the highest mean value on every index. However, as summarized in Table 1, the Friedman test, selected because the residuals for this scenario did not satisfy the normality assumption required by parametric repeated-measures ANOVA, indicates that the differences rank among all five methods are not statistically significant across all six external validity indices $\left(R_{\mathrm{SFKM}}=R_{\mathrm{BKMD}}=R_{\mathrm{Complete}}=R_{\mathrm{Ward}}=R_{\mathrm{ResConeK}}\right)$. This finding suggests that, under a relatively simple three-cluster structure, all five methods are able to produce partitions with comparable agreement with the underlying true cluster structure.

**Table 1:** Summary of the test results of six external criteria indices between grouping methods.

The number of clusters	Test Statistics	External Indices	Conclusion
k=3,k=4	Friedman & Post-hoc Nemenyi	RI, ARI, HSS, JS, FMI, RTI	$R_{\mathrm{SFKM}}=R_{\mathrm{BKMD}}=R_{\mathrm{Complete}}=R_{\mathrm{Ward}}=R_{\mathrm{ResConeK}}$
k=7	RM ANOVA & Post-hoc Bonferroni	RI, ARI, HSS, JS, FMI, RTI	$\mu_{\mathrm{SFKM}}<\mu_{\mathrm{BKMD}}<\mu_{\mathrm{Complete}}=\mu_{\mathrm{Ward}}=\mu_{\mathrm{ResConeK}}$

In the second scenario, four groups of 150 observations each were created and measured on three numeric variables. The first two groups, exhibiting circular patterns, were generated from normal distributions with a standard deviation of 50 and centers at (600, 10, 0) and (10, 0, 600), respectively. The third and fourth groups, exhibiting elongated patterns, were drawn from normal distributions with a standard deviation of one and centered at (–0.5, 0, 0.5). For the first elongated pattern, the random values for each variable were shifted by adding 160 and then ordered from 1 to 150; for the second elongated pattern, the values were shifted by adding 480 and likewise ordered from 1 to 150. An illustration of this second artificial data scenario is shown in Fig. 4. The average external validity index of 50 trials for the four-group artificial data is shown in Fig. 5.

**Fig. 4:**
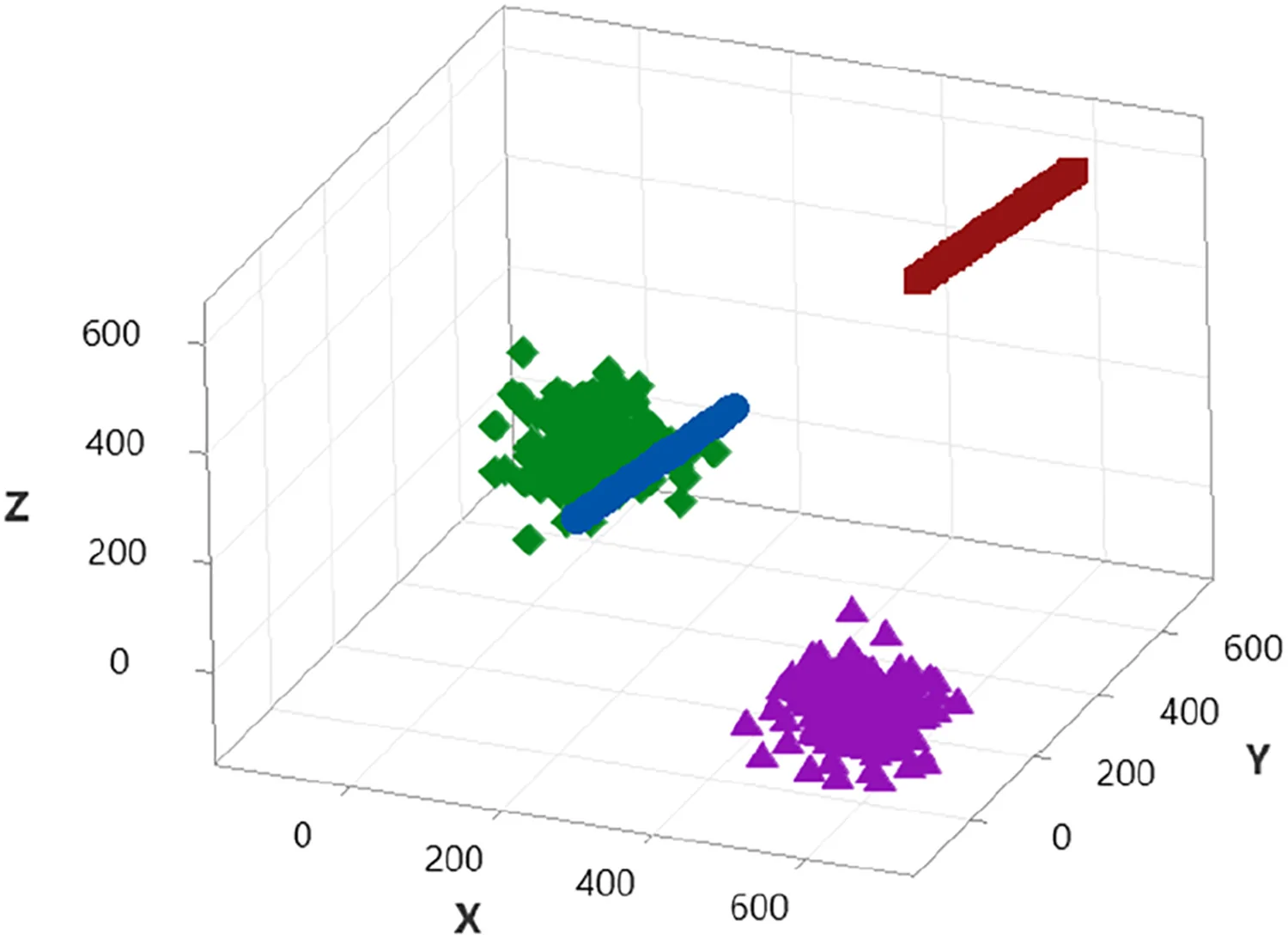
Four-groups artificial data.

**Fig. 5:**
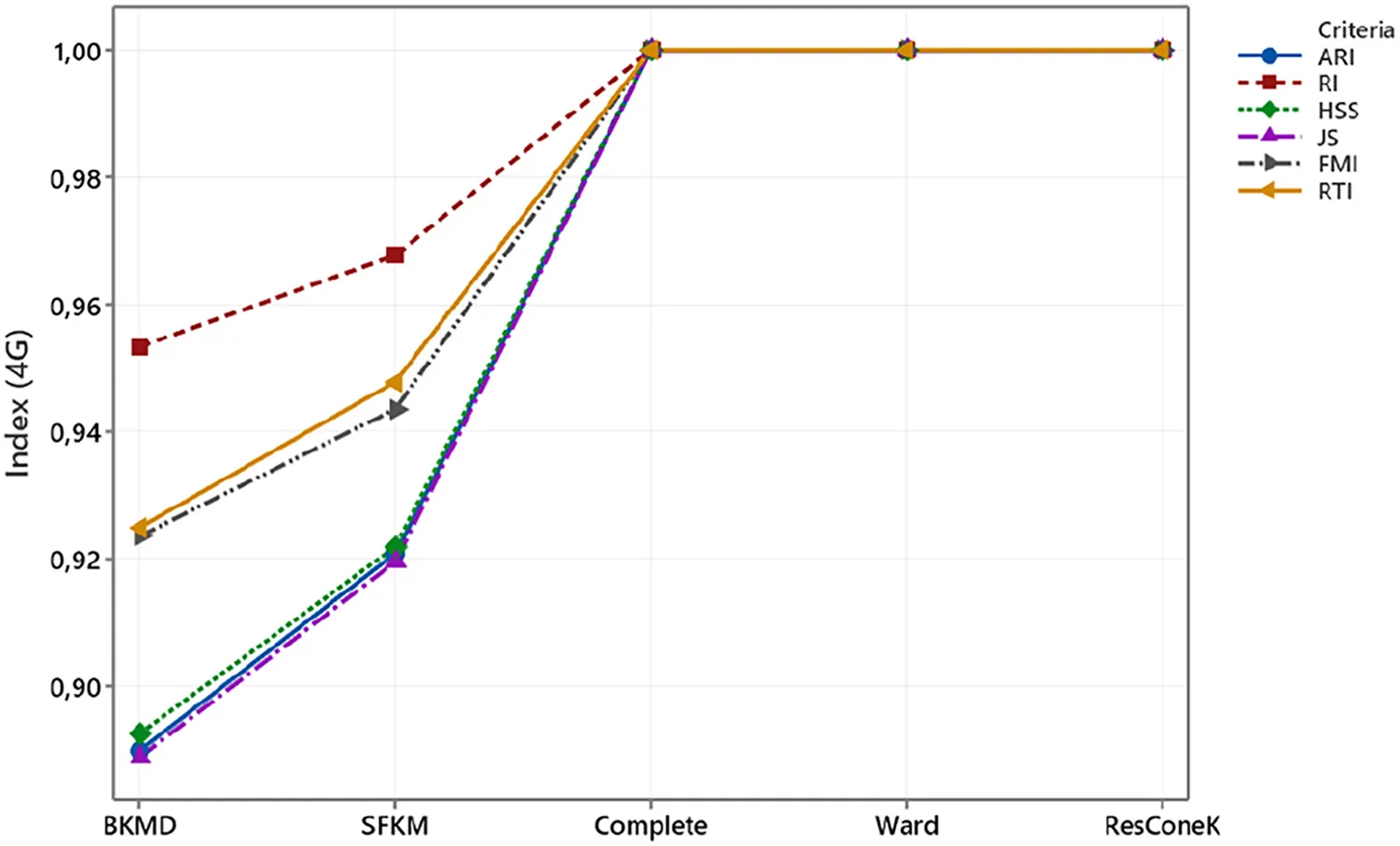
Comparison of external validity indices of four-group artificial data.

Based on Fig. 5, it can be observed that the performance of the new ResConeK method is comparable to Ward’s method and the hierarchical method with complete linkage, achieving perfect scores across all indices. In contrast, the SFKM and BKMD methods perform below these three. The Friedman test indicates that an overall difference is detected by the omnibus test. However, the magnitude of the difference between each pair is not large enough to be identified as statistically significant after pairwise comparisons and adjustment for multiple comparisons, as shown in Table 1. Thus, the k=4 result indicates that increasing complexity of the cluster structure begins to differentiate the performance patterns of the methods, although the available statistical evidence is insufficient to attribute the overall difference to a specific pair of methods.

In the third artificial data scenario, seven groups were designed, with each object measured on two numeric variables. Each group was generated from a continuous uniform distribution. For the first variable, the groups were generated within the following ranges: (5,10), (2,6), (3,7), (6,10), (10,15), (13,18), and (9,16). For the second variable, the ranges were: (0,5), (5,10), (10,15), (14,18), (12,17), (7,11), and (1,6), respectively. An example of this artificial data scenario with seven groups is presented in Fig. 6. The average external validity index of 50 trials for the seven-group artificial data is shown in Fig. 7.

**Fig. 6:**
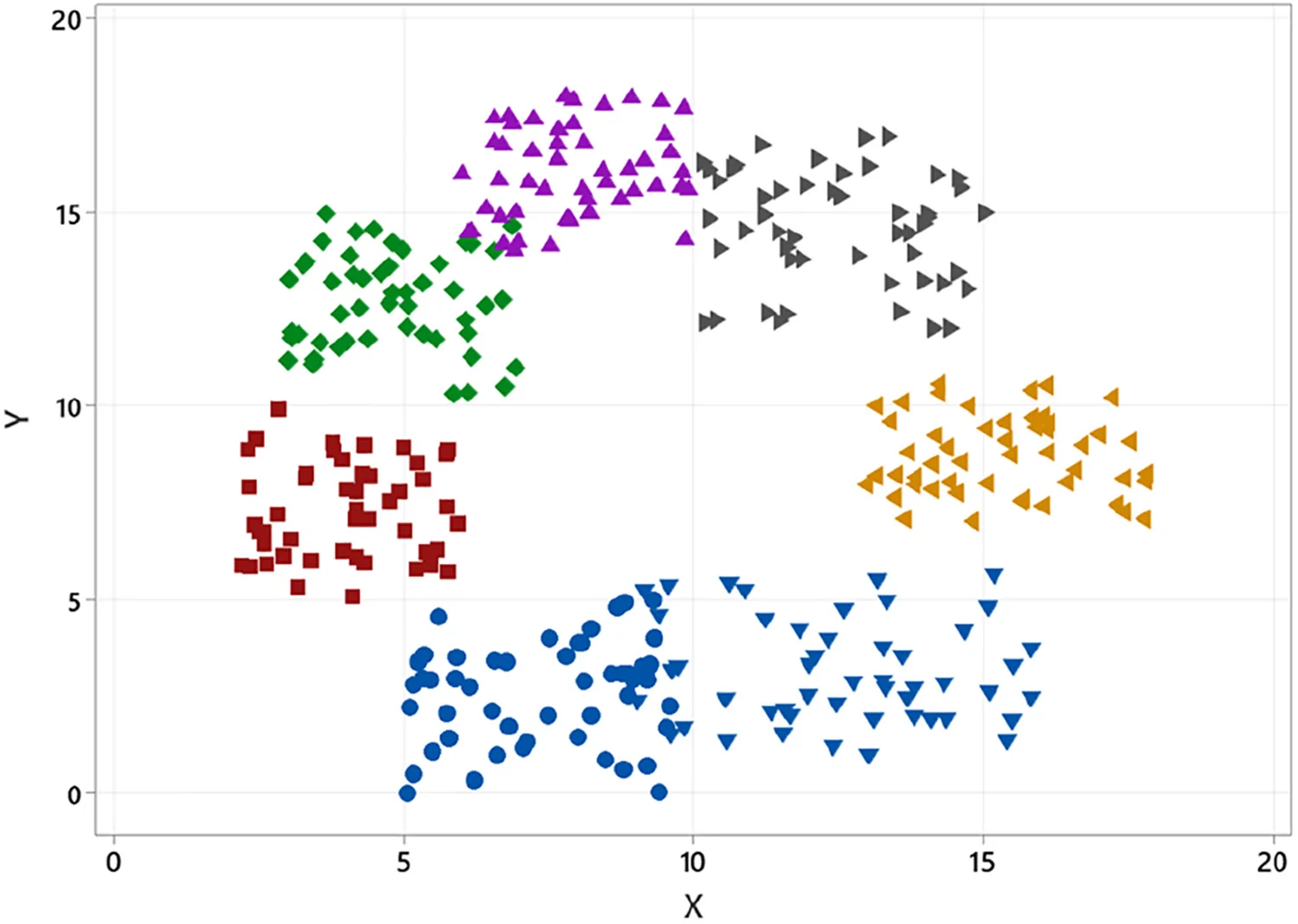
Seven-group artificial data.

**Fig. 7:**
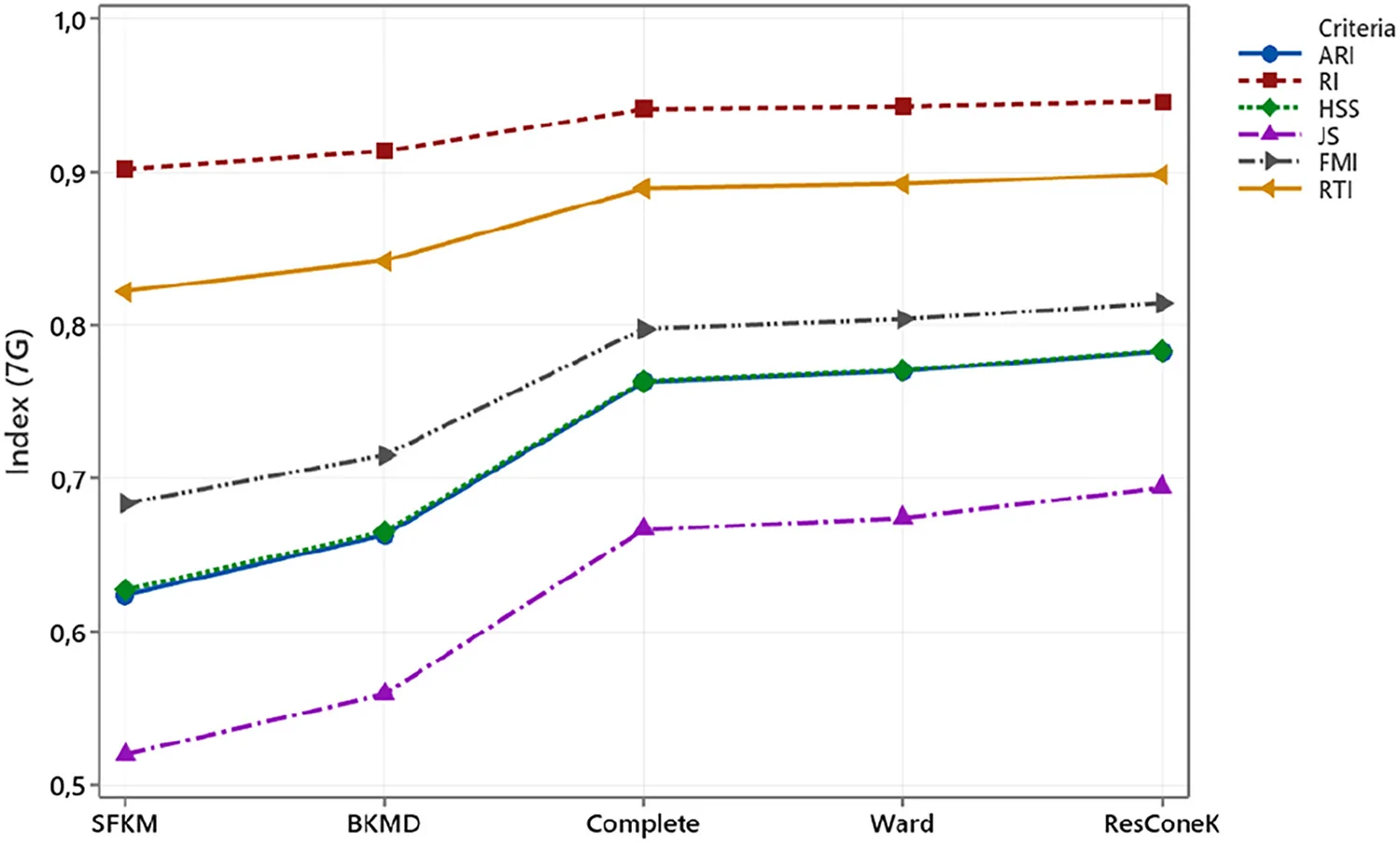
Comparison of external validity indices of seven-group artificial data.

Fig. 7 shows that none of the five clustering methods achieved a score of 0.95 for the dataset pattern presented in Fig. 6. Fig. 7 presents the mean values of the six external validity indices with methods ordered from lowest to highest overall performance: SFKM, BKMD, Complete-linkage, Ward's method, and ResConeK. A clear, monotonically increasing pattern is visible across all six indices: SFKM records the lowest mean scores throughout, BKMD shows a modest improvement over SFKM, and Complete-linkage, Ward's method, and ResConeK form a visibly higher-performing cluster, with ResConeK attaining the highest or near-highest mean value on most indices. In contrast to the three and four clusters scenario, all assumptions required for Repeated Measures Analysis of Variance (RM ANOVA) were satisfied, allowing the use of the parametric analysis. The analysis indicated significant differences among the clustering methods, and the subsequent Bonferroni post-hoc comparisons identified the specific differences. As summarized in Table 1, RM ANOVA indicates a statistically significant difference among the five methods across all six indices $\left(\mu_{\mathrm{SFKM}}<\mu_{\mathrm{BKMD}}<\mu_{\mathrm{Complete}}=\mu_{\mathrm{Ward}}=\mu_{\mathrm{ResConeK}}\right)$. Thus, ResConeK belonged to a statistically higher-performing group than SFKM and BKMD, while its performance was statistically comparable to that of complete linkage and Ward.

Overall, the evaluation of the new algorithm using three experimental scenarios reveal an interesting progression in the statistical differentiation of clustering performance. At k=3, no significant difference is detected among the methods. At k=4, an overall difference emerges according to the Friedman test, but no specific pairwise difference can be identified by the Nemenyi procedure. At k=7, the differences become sufficiently pronounced to be identified through Bonferroni-adjusted pairwise comparisons. This pattern suggests that the increasing number of clusters is associated with increasingly distinguishable performance patterns among the competing clustering methods. ResConeK demonstrates relatively stable and competitive performance across the artificial-data scenarios, with its relative advantage becoming more statistically identifiable as the number of clusters increases. These findings provide empirical support for the potential of ResConeK in clustering problems involving more complex cluster structures.

### Real-world datasets

The final clustering results produced by different methods were influenced by the data preprocessing steps. Variations in standardization techniques and the choice of distance measures between objects can lead to different clustering outcomes. The wine dataset consists of 178 objects with 13 very different variable scales. For example, the hue variable ranges between 0.48 and 1.71, or the proline variable ranges between 278 and 1680. Therefore, the wine dataset was standardized using fractional ranking. Meanwhile, the heart disease dataset has variables with mixed types (categorical and numeric). Therefore, Min-Max standardization is performed on numeric variables. The proximity measure used for the primary tumor category dataset and the small soybean dataset is the Hamming distance. For dataset with numeric variables, namely Wine, Iris, and Breast Cancer, the Euclidean distance is applied. Meanwhile, for the mixed Heart Disease data, the Gower distance measure is used, as summarized in Table 2.

**Table 2:** The profile of datasets, pre-processing and proximity measure.

No	Datasets	The number of				Pre-processing	Proximity measure (distance)
		Clusters	Object	Categorical data	Numerical data		
1.	Breast Cancer	2	683	-	9	-	Euclidean
2.	Primary Tumor	4	65	17	-	-	Hamming
3.	Heard Disease	2	303	8	5	Min-Max	Gower
4.	Iris	3	150	-	4	-	Euclidean
5.	Wine	3	178	-	13	Fractional rank	Euclidean
6.	Soybean Small	4	47	35	-	-	Hamming

In the Breast Cancer and Primary Tumor dataset, there are a number of identical or similar objects that impact the first smallest k indicators of the SFKM method is identical objects. This causes the SFKM method to fail to partition the two datasets into the group size that should be formed. Therefore, in this study specifically for these two datasets, the SFKM method is not used. Based on these considerations, a comparison of six external validation indices in the Breast Cancer, Primary Tumor, and Heart Disease dataset is presented in Fig. 8. For the Iris, Grape, and Small Soybean datasets, a comparison of the six external validation indices is shown in Fig. 9.

**Fig. 8:**
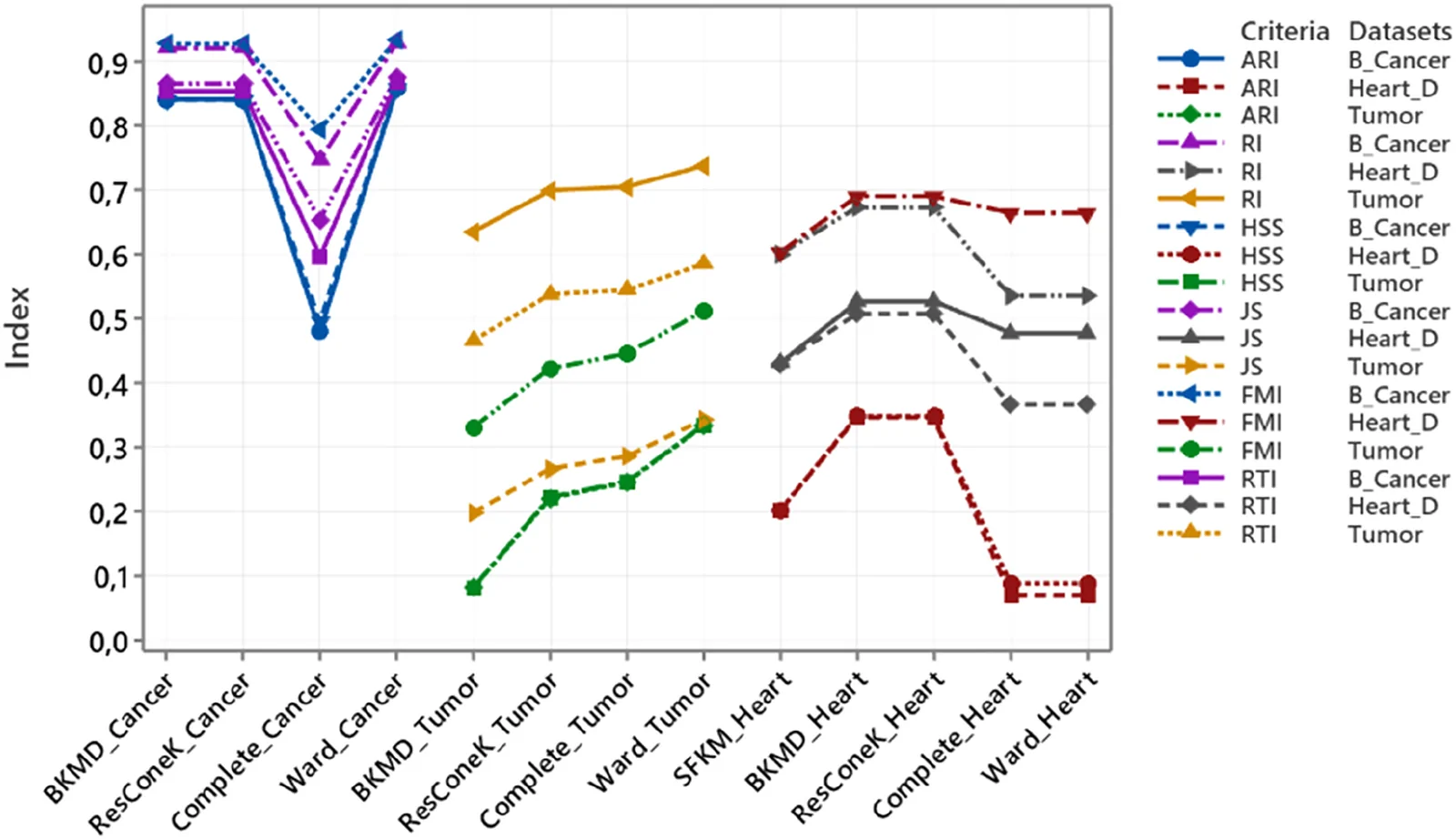
Comparison of External criteria index on cancer, tumor, and heart disease datasets.

**Fig. 9:**
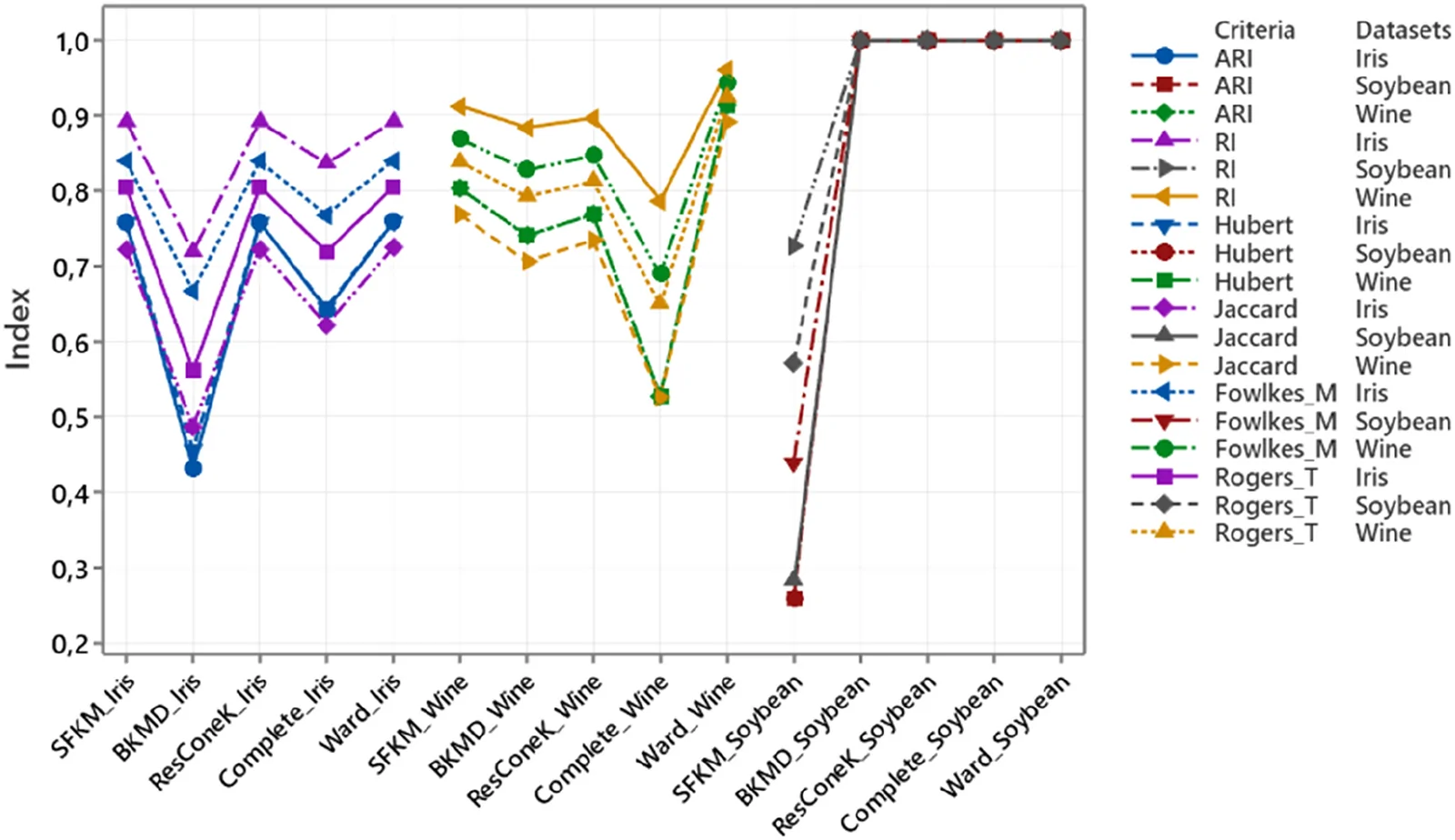
Comparison of external criteria index on iris, wine, and soybean datasets.

Fig. 8 indicates that the performance of the new ResConeK algorithm on the Breast Cancer was comparable to that of the Ward and BKMD methods. For primary tumor data, all clustering methods yielded a criterion below 0.8. This dataset presents unique challenges for clustering, given its unbalanced group composition and the presence of significant missing data. ResConeK performed relatively better than BKMD, although still below the Complete and Ward methods. As with the primary tumor data, the heart disease data also did not produce an index above 0.8. This indicates that the clustering methods used in these two datasets did not produce satisfactory groupings. For the Heart Disease dataset, the performance of ResConeK is similar to BKMD and both are above the other three methods.

Based on Fig. 9, the new ResConeK method demonstrated performance comparable to the Ward method for the Iris and Soybean Small datasets. On these two datasets, Ward consistently produced partitions with higher or comparable clustering accuracy relative to the other methods. ResConeK also succeeded in improving the performance of the BKMD method across all three datasets.

A more detailed analysis of within-group homogeneity produced by the five methods (except for Breast Cancer and Primary Tumor, which only used four methods) on these six datasets is presented in Fig. 10, covering (a) Breast Cancer, (b) Primary Tumor, (c) Heart Disease, (d) Iris, (e) Wine, and (f) Soybean Small. Fig. 10 shows that the within-group deviation (SDW(k)) is relatively consistent for ResConeK, i.e., comparable or relatively more homogeneous than the other methods.

**Fig. 10:**
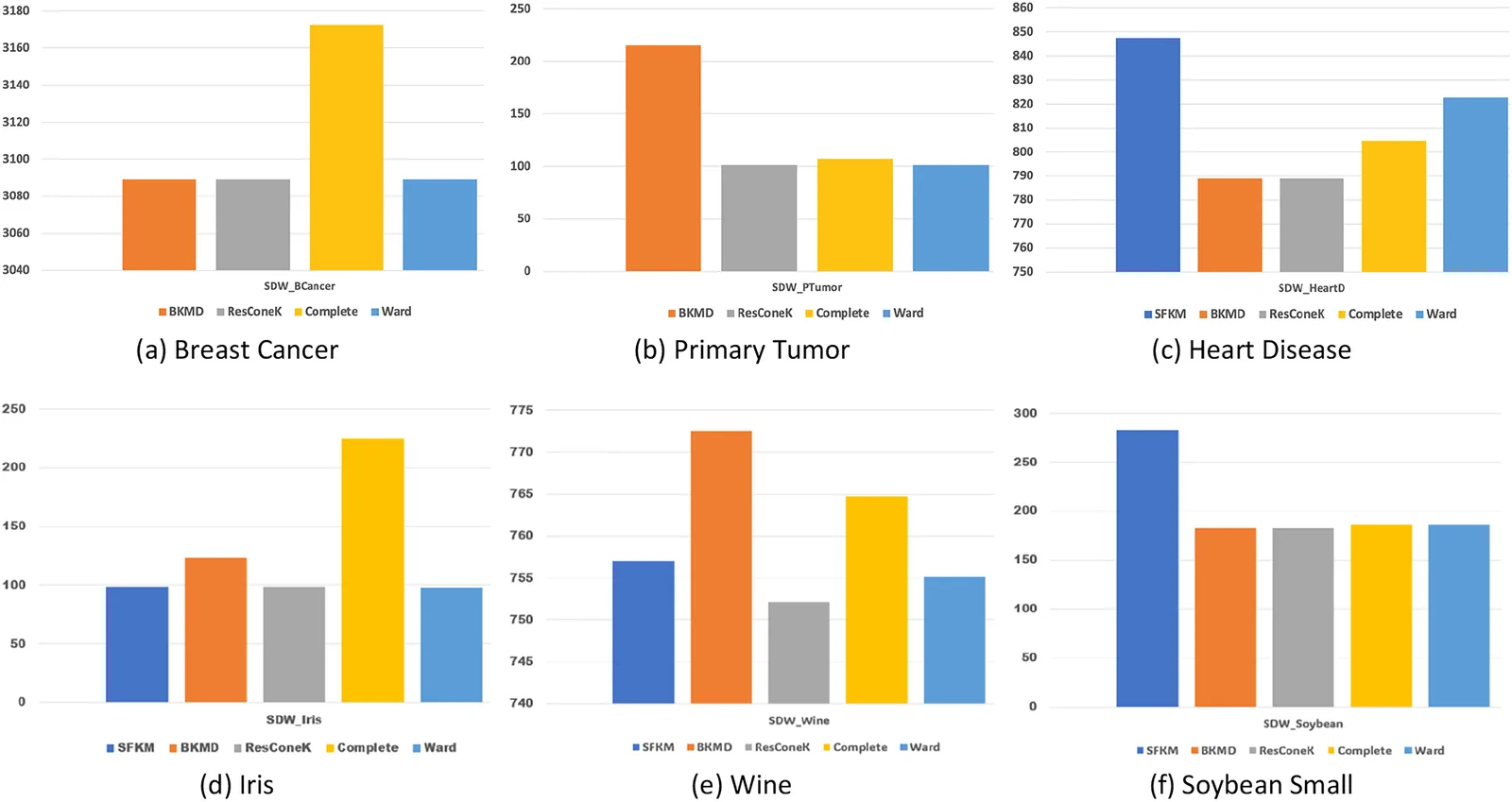
A comparison of within-group homogeneity of five methods on six real-world datasets.

Validation of the ResConeK method in terms of the suitability of clustering results using artificial data tends to improve as the group size increases. The Friedman test for the six external validation indices, Rand Index, Adjusted Rand Index, Hubert Similarity Statistic, Jaccard Similarity, Fowlkes–Mallows Index, and Rogers–Tanimoto Index, were statistically similar across three groups and different across four. The average test of the six criteria across seven groups using repeated measures ANOVA showed statistically significant differences. Post-hoc tests using the Bonferroni method also concluded that the ResConeK method performed comparable to the Ward and Complete methods, and that the average of all three was higher than the BKMD and SFKM methods. In addition, validation using six real-world datasets, namely Breast Cancer, Primary Tumor, Heart Disease, Iris, Wine, and Soybean Small shows that, when partitioned based on the number of cluster references for each dataset, the clusters formed by ResConeK are comparable or relatively more homogeneous than other methods.

An important result of this study is that the resampling strategy used in the ResConeK method to determine the final medoids provides an alternative mechanism to reduce the dependence of clustering solutions on a single initial medoid configuration. This approach can help mitigate the risk of local optimization, especially in datasets containing a large number of identical objects.

### Limitations

The proposed ResConeK algorithm enhances clustering quality by incorporating a resampling strategy into block-based k-medoids. Future work will focus on strengthening the theoretical foundations of ResConeK, particularly by investigating its sensitivity to parameter settings, and on extending its applicability to high-dimensional data through the integration of dimensionality reduction techniques.

## Ethics statements

This study did not involve human participants, animal subjects, or social media data. All analyses were performed using artificially generated datasets and publicly available datasets from the UCI Machine Learning Repository. Therefore, no ethical approval or informed consent was required.

## CRediT author statement

**Kariyam:** Conceptualization, Methodology, Investigation, Writing – Original Draft, Editing and Visualization, Funding acquisition.

**Feri Wijayanto:** Software, Visualization, Writing - Review & Editing, Investigation, Formal analysis.

**Edy Widodo:** Data curation, Project Administration, Validation and Supervision.

## Supplementary material *and/or* additional information

The R scripts used for the implementation of the proposed ResConeK algorithm and the analyses presented in this study are available at the following link:

ResConeK R Code: https://bit.ly/ResConeK_V0

The code is provided to facilitate the reproducibility of the proposed method and the analyses reported in this study.

## Declaration of competing interest

The authors declare that they have no known competing financial interests or personal relationships that could have appeared to influence the work reported in this paper.

## Data Availability

No data was used for the research described in the article.
